# The system’s genetics of depression and its somatic and mental comorbidities

**DOI:** 10.1515/tnsci-2022-0229

**Published:** 2022-07-26

**Authors:** Liubov S. Kalinichenko, Johannes Kornhuber, Christian P. Müller

**Affiliations:** Department of Psychiatry and Psychotherapy, University Clinic, Friedrich-Alexander-University of Erlangen-Nuremberg, Schwabachanlage 6, 91054 Erlangen, Germany; Centre for Drug Research, Universiti Sains Malaysia, 11800 Minden, Penang, Malaysia

**Keywords:** major depressive disorder, comorbidities, shared genetic mechanisms, somatic diseases, mental disorders

## Abstract

Depression is a common mood disorder characterised by high comorbidity with other mental and somatic diseases. New studies reveal a shared genetic base for mental core symptoms and somatic comorbidities. Functional analyses showed multiple brain–body pathways involved. This may help considering new therapeutic approaches for depression as a system’s disorder.

Major depression (MD) is one of the most common mental disorders with high social and economic impact. About 4.4% of the world’s population is affected by MD, which determines significant costs for treatment of this mood disorder [1]. Despite the pronounced medical and social significance of MD, there is no clear theory unifying the molecular and genetic mechanisms of it. The genetic basis of depression is complicated due to high etiological and phenotypic heterogeneity. The genetic structure of MD comprises a large number of genetic loci, which might induce various phenotypic effects and display complex interactions [2]. This allows to suggest that the genetic background of this mental disorder is of specific interest due to possible associations with under-researched molecular and cellular pathways.

Besides the mental and behavioural core symptoms, the vast majority of patients with MD develop co-occurring or comorbid disorders. Multiple epidemiological data indicate comorbidity of depression with other brain disorders, particularly anxiety, substance use disorder, Alzheimer disease, and epilepsy [3,4]. MD is also associated with several peripheral disorders, such as cardiovascular diseases, atherosclerosis, obesity, diabetes mellitus, some types of cancer, immune diseases, migraine, irritable bowel syndrome (IBS), and musculoskeletal diseases. For some of these disorders, the casual relationship with MD has not been observed yet, thus they can be considered as co-occurring. However, for many of them, such as MD and alcohol use disorder, overlaps in genetic and molecular mechanisms were observed, thus allowing to suggest this interaction as comorbidity. Comorbid MD aggravates the course of a physical disease resulting in higher degree of functional impairment and disability. This is associated with increased service utilisation and higher medical costs [4,5]. On the other hand, MD may develop as a consequence of peripheral disorders, such as pain syndromes, stroke, skin diseases, cancer, and osteoporosis [5,6].

Two new studies are the first to show bidirectional genetic mechanisms determining the development of comorbid depression and somatic disorders. A genome-wide analysis on 53,400 patients and 433,201 controls revealed strong genetic correlations between IBS and mood disorders, such as anxiety, depression, insomnia, and neuroticism [7]. These disorders are more likely to share pathogenic pathways due to the genetic overlap rather than affective disorders causing the IBS. At least four genes implicated in the pathogenesis of IBS affected mood disorder development. They include *NCAM1*, encoding neural cell adhesion molecule 1, and *CADM2*, encoding cell adhesion molecule 2. These genes regulate neural circuit formation and affect white matter microstructure in the brain. Further identified genes include *PHF2/FAM120A*, coding PHD (plant homeodomain) finger protein 2 from the family with sequence similarity 120A, and the dedicator of cytogenesis 9, *DOCK9*. These genes play a key role in brain development. Interestingly, predominant brain expression of these genes proposes the central nervous system as the main site where these gene variants exert their action determining the association between IBS and depression and anxiety [7]. However, peripheral mechanisms of the comorbidity based on the expression of the genetic variants outside the brain cannot be excluded. Therefore, shared genetic risk pathways between anxiety and depression and IBS might be independent of the comorbidity between these central and peripheral disorders [9].

In another study, the genetic mechanisms of a comorbidity trias alcohol abuse-depression/anxiety-bone disorder are discussed. At the clinical level, the comorbidity between depression, anxiety, and substance use disorder is of specific interest due to very high prevalence [8]. Alcohol use is often established to self-medicate for depression and anxiety symptoms [9], but may aggravate to a disorder state, thus leading to a vicious circle of negative emotional states [8]. A genetic association analysis in 456,693 volunteers found an association of numerous haplotypes of the *SMPD3* gene, coding for the enzyme neutral sphingomyelinase-2 (NSM), with alcohol consumption, depression, and anxiety [10]. A functional analysis in mice confirmed a crucial role of NSM in the control of emotional state, alcohol consumption, and their interaction by a regulation of hippocampal volume development, cortical connectivity, and monoaminergic responses in the brain. Moreover, clinical data revealed significant associations between *SMPD3* haplotypes and total bone mineral density of the left and right femurs in humans. Thus, *SMPD3* contributes to the association between negative mental states and alcohol use and altered bone mineralisation. NSM was shown to functionally control the bone–brain communication by enhancing osteocalcin signalling. Osteocalcin, released from the osteoblasts into the blood, crosses the blood–brain barrier and reaches distinct receptor targets in the brain. By this way, it can independently suppress alcohol consumption and reduce depressive behaviour in mice [10]. These findings suggest a strong link between the natural variance in the *SMPD3* gene with alcohol use disorder, emotional behaviour, and bone mineral density. As distinct from the previous study, the effects of *SMPD3* gene involved molecular mechanisms both in the central nervous system and peripheral systems, and their interaction. Altogether, a single genetic base was found to modulate multiple pathways, which interlink the symptoms of the mental–physical comorbidity trias of alcohol abuse-depression/anxiety-bone disorder.

These studies expand the view on the comorbidities between mental and somatic disorders and open a principally new perspective on shared genetic risks across distinct syndromes of MD. The strong interconnection of depression with other mental and peripheral disease symptoms may no longer be seen as simple co-occurrences or comorbidities, but as multiple bidirectional pathways with a shared genetic base (Figure 1). It should be mentioned, that certain environmental factors might also affect the development of comorbid MD and other disorders, particularly alcohol use disorder, but the genetic base has slightly more influence on comorbidity [11]. Considering the extensive association of depression with several life-threatening disorders, the identification of new shared genetic bases might improve the understanding of the disorder and its pathogenetic pathways to a system’s event that always develops as an interaction between central and peripheral processes. This might change diagnostic and therapeutic approaches for MD treatment. MD may be considered as a central health care problem, which requires interdisciplinary treatment strategies. To enhance therapeutic efficacy of peripheral disorders and life quality of patients, the diagnostics and therapy of somatic disorders may not only include a screening for depression, but also for potentially shared genetic risk factors.

**Figure 1:**
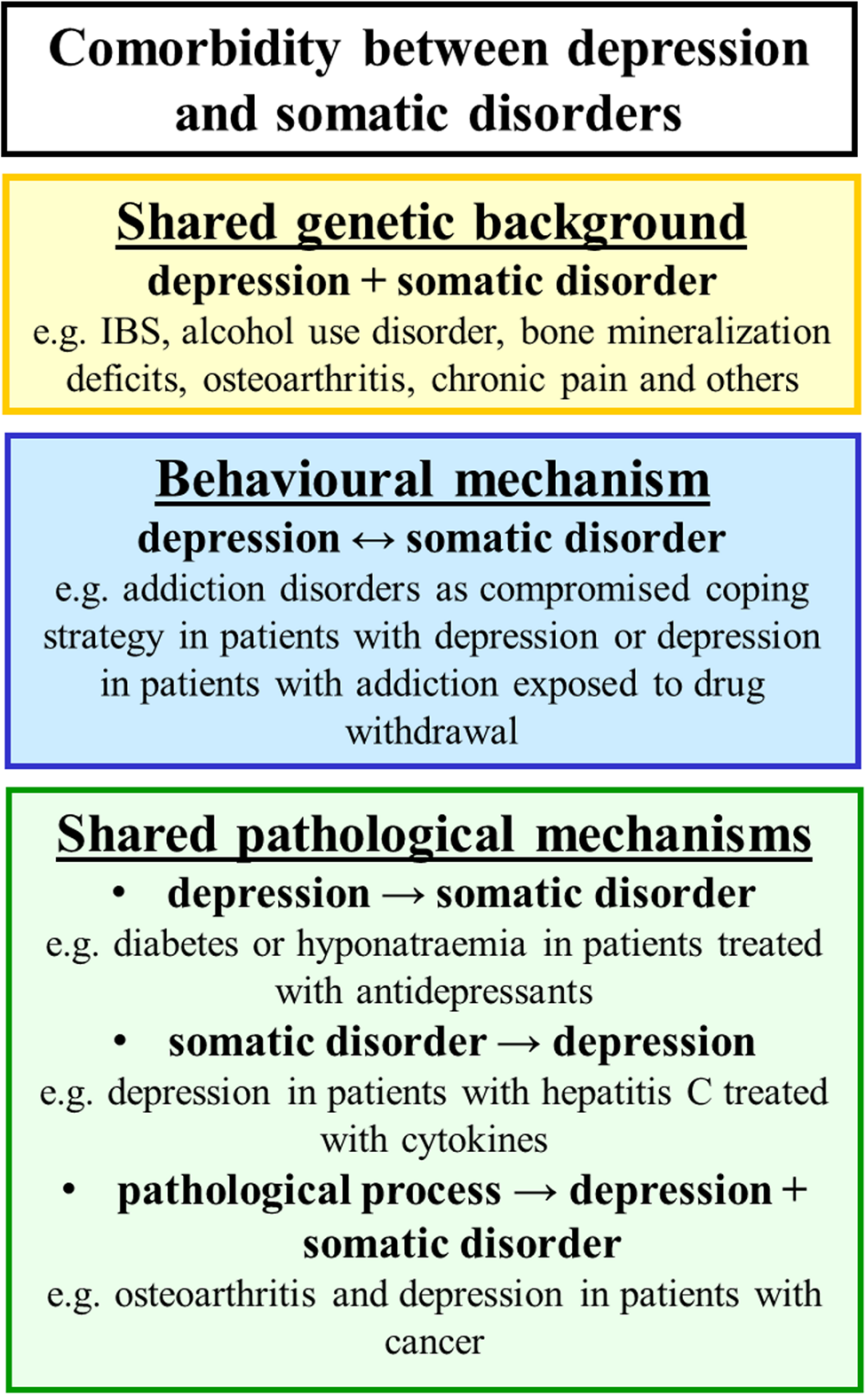
Main pathological mechanisms underlying comorbidity between MD and somatic and mental disorders. Modified from ref. [5].
